# Health‐related quality of life and medication adherence of people living with epilepsy in Pakistan: A cross‐sectional study

**DOI:** 10.1002/brb3.3127

**Published:** 2023-07-29

**Authors:** Maria Tanveer, Azhar Hussain Tahir, Mansoor Iqbal, Faiza Aslam, Ali Ahmed

**Affiliations:** ^1^ Department of Pharmacy Quaid‐I‐Azam University Islamabad Pakistan; ^2^ Primary and Secondary Healthcare Department Government of Punjab Lahore Pakistan; ^3^ Neurology Department Pakistan Institute of Medical Sciences (PIMS) Islamabad Pakistan; ^4^ Department of Psychiatry Rawalpindi Medical University Rawalpindi Pakistan; ^5^ Riphah Institute of Pharmaceutical Sciences Riphah International University Islamabad Pakistan; ^6^ Monash University Health Economics Group (MUHEG) School of Public Health and Preventive Medicine Monash University Melbourne Australia

**Keywords:** adherence, epilepsy, low‐middle‐income country, Medication Adherence Rating Scale (MARS), Pakistan, quality of life (QOL), Quality of Life in Epilepsy‐31 (QOLIE‐31), seizures

## Abstract

**Introduction:**

The primary purpose of this study was to determine adherence and health‐related quality of life (HRQoL) in PWE. Secondary aims were to assess association between adherence and HRQoL and determine predictors of HRQoL in PWE in Pakistan.

**Methods:**

A descriptive cross‐sectional study was conducted among PWE receiving treatment from two tertiary care hospitals of Pakistan. The HRQoL and adherence were assessed with Urdu versions of Quality of Life in Epilepsy‐31 (QOLIE‐31), and Medication Adherence Rating Scale (MARS). Relationship between HRQoL and adherence was assessed by Pearson's product‐moment correlation coefficient. Forced entry multiple linear models were used to determine relationship of independent variables with HRQoL.

**Results:**

219 PWE with a mean (±standard deviation) age, 34.18 (± 13.710) years, participated in this study. The overall weighted mean HRQoL score was (51.60 ± 17.10), and mean score for adherence was 6.17 (± 2.31). There was significant association between adherence and HRQoL in PWE (Pearson's correlation = 0.820–0.930; *p* ≤ .0001). Multiple linear regression found adherence (*B* = 16.8; *p* ≤ .0001), male gender (*B* = 10.0; *p* = .001), employment status (employed: *B* = 7.50; *p* = .030), level of education (Tertiary: *B* = 0.910; *p* = .010), duration of epilepsy (>10 years: *B* = –0.700; *p* ≤ .0001), and age (≥46 years: *B* = –0.680; *p* ≤ .0001), and ASM therapy (polypharmacy: *B* = 0.430; *p* = .010) as independent predictors of HRQoL in PWE from Pakistan.

**Conclusions:**

The findings suggest PWE from our center have suboptimal adherence which affects HRQoL. Independent factors such as male gender, employment status and duration of epilepsy are predictors of HRQoL.

## Graphical abstract

Epilepsy is a common chronic neurological disorder with a huge treatment gap, especially in low and middle‐income countries like Pakistan. The treatment gap is thought to affect adherence and HRQoL in PWE.

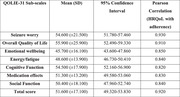

## 1 INTRODUCTION

Epilepsy is a common chronic neurological disorder affecting 50–70 million people worldwide (Geneva: World Health Organization, 2019; World Health Organization, 2016), with 80% of these living in low‐ and middle‐income countries (LMICs), and more than three‐quarters do not receive treatment (Espinosa‐Jovel et al., 2018; World Health Organization, 2016). With proper diagnosis and anti‐seizure medications (ASMs) therapy, up to 70% can live a seizure‐free life (Geneva: World Health Organization, 2019). With more than 5 million new cases every year, epilepsy accounts for 0.7% of the global disease burden (Espinosa‐Jovel et al., 2018) and 13 million disability‐adjusted life years (DALYs) (Geneva: World Health Organization, 2019; World Health Organization, 2016) and it is the 36th leading cause of DALYs (Espinosa‐Jovel et al., 2018). In Pakistan, recent epidemiological data are unavailable but prevalence is estimated to be 9.99/1000, accounting for 2 million people and 1/10th of the global epilepsy burden (Siddiqui et al., 2015). Here, the incidence rate is higher in adults under the age of 30 (World Health Organization, 2016), which may impair their key developmental areas like autonomy, independence, and social development (Chew et al., 2019). Despite recent advances in treatment strategies for epilepsy and increased global awareness, the treatment gap, societal stigmatization, and discrimination continue to adversely affect HRQoL in persons with epilepsy (PWE) (Skogen et al., 2023) like in other chronic diseases such as cancer (Götze et al., 2018), HIV (Rzeszutek et al., 2021; Skogen et al., 2023), and psoriasis (Pereira et al., 2012). PWE like other chronic diseases requires life‐long care and determining HRQoL becomes a vital point of care making it essential to determine factors that influence quality of life (Skogen et al., 2023).

Health‐related quality of life (HRQoL) is defined as “those aspects of self‐perceived well‐being that are related to or affected by the presence of disease or treatment” (Karimi & Brazier, 2016). Persons with epilepsy (PWE) often have HRQoL significantly lower than those with other chronic diseases like hypertension and diabetes (Michaelis et al., 2018). PWE in Pakistan report facing problems in getting education (Najam‐Us‐Sahar & Chaudry, 2010), employment (Aziz & Akhtar, 1997), marriage (Lalwani et al., 2015), and societal acceptance (Sayed, 2014), which are some of the challenges affecting their HRQoL. Moreover, patients have to bear financial burden of epilepsy which not only affect themselves but also their families as they have to bear the cost of disease diagnosis and treatment (Najam‐Us‐Sahar & Chaudry, 2010). A recent study reports the annual mean cost of treatment of epilepsy in Pakistan is Rs. 9004.9 ($55.3) for levetiracetam, Rs. 4545.5 ($27.9) for valproic acid, Rs. 3224.2 ($19.8) for carbamazepine, and Rs. 14075.5 ($86.5) for lamotrigine (Khan et al., 2021). In terms of treatment cost‐effectiveness, epilepsy is ranked in the top five of all noncommunicable diseases by the world bank (WB) (Chisholm & Saxena, 2012), but despite this, the treatment gap in Pakistan is enormous, with only 28.5% in urban and 1.90% in rural areas receiving treatment (World Health Organization, 2016).

Adherence is the “extent to which the patient's behavior matches agreed recommendations from the prescriber” (Chakrabarti, 2014). In LMICs especially Pakistan, limited health resources and inequities in healthcare access (World Health Organization, 2016), low socioeconomic status, low education (Govil et al., 2021; Siddiqui et al., 2015), limited and nonavailability of ASMs, duration and cost of treatment and religious beliefs are the main obstacles impacting adherence to ASM therapy (Sayed, 2014; Siddiqui et al., 2015). Studies show lack of adherence increases mortality three folds (Mcauley et al., 2015; Zheng et al., 2019), compromises HRQoL, impacts important treatment outcomes, and escalates the overall health care costs (Pakpour et al., 2015).

In Pakistan, there is only one neurologist for every 1.4 million people (Siddiqui et al., 2015). Most people, despite being educated, seek first line treatment from faith healers, Hakims, peers, and ayurvedic drugs (Sayed, 2014; Siddiqui et al., 2015; World Health Organization, 2016) because of myths and superstitions associated with the disease in Pakistan (World Health Organization, 2016). Many have not heard of epilepsy before being diagnosed and most consider it a cause of sins, super natural powers, black magic (Najam‐Us‐Sahar & Chaudry, 2010; Usman, 2019) and believe it is contagious (Sayed, 2014; Usman, 2019). Most report smelling a shoe as the most frequent way to control an epileptic seizure while others report putting keys in hands and contacting a faith healer (Sayed, 2014).

Epilepsy prevalence pattern and distinct cultural environment in Pakistan (World Health Organization, 2016) impacts adherence and HRQoL in ways unique to this population. With the advent of newer ASM therapies and more restricted healthcare resources, the need for patient satisfaction measurement is now more significant (Brusturean‐Bota et al., 2013). In Pakistan, a study was conducted in 2010 to evaluate HRQoL in PWE on a small sample size (*n* = 50) (Najam‐Us‐Sahar & Chaudry, 2010) but no study has explored association between these variables or assessed predictors within this population, thus a baseline study is necessary to inform health authorities to design culturally adapted evidence‐based interventions.

### 1.1 Current study

Taking into consideration the aforementioned research gaps, the primary aim of our study was to assess HRQoL, by using QOLIE‐31, and adherence by using MARS, in PWE. The secondary aim was to evaluate the association between HRQoL and adherence and determine the predictors of HRQoL in PWE from our center.

## 2 METHOD

### 2.1 Study design and setting

This cross‐sectional study was conducted from July 2019 to January 2020 at neurology departments of two institutions; Pakistan Institute of Medical Sciences (PIMS), Islamabad, and District Health Quarter (DHQ) Hospital, Rawalpindi, Pakistan. The Strobe checklist was used to report the study by following the guidelines by Equator network. PIMS is the largest hospital in the region with a specialized 24‐bed care unit for epilepsy patients equipped with the latest neurodiagnostic facilities. The PIMS outdoor patient department (OPD) serves 80–100 patients free of cost daily, covering diagnostics and medications, and the DHQ hospital serves 60–80 patients in OPD each day (Ahmed et al., 2021). The institutes serve people of various ethnicities and socioeconomic backgrounds (Ahmed et al., 2021).

### 2.2 Eligibility criteria

Adult PWE (aged ≥18 years) with physician confirmed diagnosis (according to International League of Epilepsy criteria 2018), who were on ASMs for at least 6 months and were adapt to conversing and understanding Urdu (Pakistan's national language) were selected for the study. However, patients were excluded if they were suffering from nonepileptic spells, had psychiatric problems, pregnant patients, drug resistant epilepsy, and had cognitive impairment or were seriously ill.

### 2.3 Sample size and sampling technique

The single population proportion formula (Charan & Biswas, 2013) was used to determine sample size for this study with the assumptions: the proportion of PWE being seizure‐free was taken from a study conducted in Pakistan in 2010 (Najam‐Us‐Sahar & Chaudry, 2010) because it gives the maximum sample size from the factors affecting HRQoL (38%, *p* = .38); this was used because recent prevalence data for epilepsy is not available from Pakistan, level of significance 5% (*α* = 0.05), *Z* (*α*/2)^2^ = 1.96, and margin of error 7% (*d* = 0.07).

$$n_{o}=Z{\left(\alpha /2\right)}^{2}\times p\;\left(1-p\right)/d^{2}$$

Hence, minimum sample size required was = (1.96)^2^ × 0.38 (1 – 0.38) / (0.07)^2^ = 185.

An adjustment of the sample for unintentional error or missing rate was performed (Sakpal, 2010):

$$n=\frac{n}{1-d},$$

where *n* = 185 and *d* = 7%. The total minimum sample size required was 199. A simple purposive sampling technique was employed and a total of 219 patients were approached and voluntarily interviewed in the study, and two hundred and nineteen patients were included in the final analysis.

### 2.4 Sociodemographic and clinical characteristics of PWE in Pakistan

The sample for current study consisted of 219 PWE. The mean age of the respondents was 34.18 with a standard deviation of ± 13.71 years. Almost half (*n* = 106, 48.4%) of them fall in the age group of 26–45 years. The mean duration of epilepsy was 5.73 years. Most of the respondents (*n* = 116, 53%) were males and majority (*n* = 168, 76.7%) were employed. Regarding marital status, most (*n* = 149, 63%) were single and one‐third (*n* = 70, 32%) were married. Regarding education (*n* = 5, 2.30%) had less than primary education, rest of the respondents completed higher levels of education. The majority of patients could afford ASM medicines 57.5% (*n* = 126) and a total of (*n* = 140, 63.9%) of patients were on monotherapy and carbamazepine (*n* = 59, 26.9%) was the most prescribed ASM and carbamazepine + valproic acid (*n* = 62, 28.0%) was the most prescribed combination (Table **1** **)**.

**TABLE 1:** Sociodemographic and clinical characteristics of PWE in Pakistan (*n* = 219).

Variable	*n* (%)
**Gender**	
Female	103 (47.0)
Male	116 (53.0)
**Age, mean (SD)**	34.2 (±13.7)
18–25	72 (32.9)
26–45	106 (48.4)
>46	41 (18.7)
**Employment status**	
Employed	168 (76.7)
Unemployed	51 (23.0)
**Marital status**	
Single	149 (68.0)
Married	70 (32.0)
**Education level**	
Less than primary education	5 (2.30)
Primary	24 (10.9)
Secondary	185 (84.5)
Tertiary	5 (2.30)
**Disease duration (in years), mean (SD)**	5.73 (±6.15)
<5	151 (68.9)
5–10	39 (17.8)
>10	29 (13.2)
**Can afford ASM medicines?**	
Yes	126 (57.5)
No	93 (42.5)
**Antiepileptic drugs**	
Monotherapy	*n* = 140, 63.9%
Carbamazepine	59 (26.9)
Levetiracetum	36 (16.0)
Valproic acid	25 (11.4)
Topiramate	10 (4.60)
Clonazepam	4 (1.80)
Lacosamide	3 (1.40)
Zonisamide	2 (0.90)
Lamotrigine	1 (0.50)
**Combination therapy**	*n* = 79, 36%
Carbamazepine + valproic acid	62 (28.0)
Lorazepam + valproic acid	10 (4.60)
Topiramate + lamotrigine	3 (1.40)
Gabapentin + topiramate	3 (1.40)
Levetiracetum + valproate sodium	1 (0.50)

### 2.5 Study instrument

The questionnaire booklet consisted of two parts: Part 1 covered the patient's sociodemographic (age, marital status, medication affordability, employment status) and clinical characteristics (disease duration, ASMs) and Part 2 included the QOLIE‐31 and MARS.

### 2.6 HRQoL

QOLIE‐31 is a disease‐specific HRQoL survey instrument for PWE aged ≥18 years consisting of 31 items divided into seven subscales, namely, *Seizure‐Worry*, *Emotional well‐being, Energy/Fatigue*, *Cognitive‐Function*, *Medication‐effects*, *Social Functioning*, and *Overall‐Quality of Life* (Quality of Life in Epilepsy Inventory (QOLIE‐89 & QOLIE‐31) | RAND, 2019). The scoring involves converting raw precoded numeric values into a scale having a range of 0–100. The higher the score, the better the quality of life of PWE. QOLIE‐31 has shown to be a reliable questionnaire for self‐assessing the HRQoL of PWE in Pakistan (Najam‐Us‐Sahar & Chaudry, 2010).

### 2.6 Medication adherence

MARS is composed of 10 items with a dichotomous response, that is, either a yes or no answer (Fialko et al., 2008). The questionnaire determines adherence through three domains: items 1–4 assess medication adherence behavior, items 5–8 assess the individuals’ attitude towards consuming medication, and items 9–10 assess adverse/side effects of drugs. The total score is in a range of 0 to 10: the higher the score, the greater the degree of medication adherence (Fialko et al., 2008). Individuals with a score ≥7 were adherent to their ASMs therapeutic regime. MARS was translated into Urdu by following the guidelines provided in Linguistic Validation Guidance of a Clinical Outcome Assessment (COA) published by Mapi Research Trust (Group et al., 2016). A group of competent postgraduate pharmacists consisting of 4 members and a neurologist from PIMS (MI), performed content and face validity for translated MARS. The version was tested in a pilot study of 35 randomly selected “stable” epilepsy patients (controlled epilepsy), and the value of Cronbach's alpha was evaluated, which was 0.74, which shows satisfactory internal consistency. For test–retest reliability, adherence was assessed after two weeks on 30 epilepsy patients. The value of the intraclass correlation coefficient of 0.75 (95% CI and *p* < .00) showed good reliability.

### 2.7 Operational definitions

#### 2.7.1 HRQoL

There is no cut off value for good or poor HRQoL as the scoring manual states the higher the mean score for a patient the better the HRQoL and this assumption was followed in present study.

#### 2.7.2 Adherence

For present study, respondents with a score ≥ 7 on MARS were regarded adherent to their ASMs therapeutic regime and a score of ≤6 as suboptimal adherence.

### 2.8 Ethical considerations

The ethical approval of the study was obtained from the Ethical Review Board of Pakistan Institute of Medical Sciences (PIMS) and Rawalpindi Medical University (RMU) for DHQ hospital, Pakistan (Approval No. 1255, 1028). The observance and maintenance of confidentiality and anonymity were practiced throughout the research. Before data collection, the respondents were informed about the purpose of the research and usage of collected data. All principles of research ethics, including the Declaration of Helsinki (Sohn, 2013) and Good Clinical Research Practice (Barton, 2007) were strictly adhered to during the study, and it was ensured that no unauthorized person had access to the collected data. Privacy of patients was ensured by making questionnaires anonymous.

### 2.9 Data collection procedure

The data collection was carried out for 7 months (from July 2019 to January 2020). The principal investigator MT and PI approached each 5th epilepsy patient in the serial list allotted to patients provided by the institutes, in the OPD of the neurology department in both institutes on their respective OPD days (PIMS: Monday, Tuesday, and Thursday; DHQ hospital: Wednesday and Saturday). Patients who agreed to participate in the study were informed about the research nature and were assessed against the predefined inclusion and exclusion criteria by MT. In order to assess the patients against inclusion/exclusion criteria, their medication records were checked. PI helped individuals with “less than primary education” participants by interviewing them verbally (asking questions verbatim) and recording their answers. The rest of the respondents were asked to fill both parts of the questionnaire independently and entirely, and if they needed any explanation regarding any item, the principal investigator provided them required assistance. It took 15–25 min for each respondent to complete the questionnaire. The principal investigator checked each filled questionnaire, and if any item was skipped, the respondent was requested to fill it. None of the patients was given monetary benefits to participate in the study.

### 2.10 Statistical analysis

The filled questionnaires were checked to ensure completeness, and then data were entered in MS Excel 2013. For statistical analysis, it was then imported to Statistical Package for Social Sciences (SPSS) version 21. The clinical and demographic characteristics were described through descriptive statistics. The categorical variables were displayed in percentages and frequencies, while for continuous variables means and standard deviations were calculated. The QOLIE‐31 scoring was performed according to the questionnaire manual provided by the RAND organization (Vickrey et al., 1993). As the QOLIE‐31 has been adapted from seven different scales, each scale has an additional coding and scoring method. The scores for seven subscales include calculating the weightage of the individual scales and summing up the seven weights gives an overall score for a particular patient (Vickrey et al., 1993). The score of MARS was calculated by summing up individual item scores (Thompson et al., 2000) and categories of adherence used are as defined above in operational definitions. The Pearson's product‐moment correlation coefficient was used to determine the association between adherence and HRQoL. The data was checked through preliminary analysis to ensure that there were no violations of linear regression model assumptions such as normality, linearity, variance equality, multicollinearity and homoscedasticity. Forced entry multiple linear models were used to determine relationship of independent variables with HRQoL while controlling for confounders. A 95% confidence interval and a *p* value of <.05 were considered statistically significant.

## 3 RESULTS

### 3.1 Reliability of study instrument

The internal consistency of the QOLIE‐31 Urdu version was ensured by determining Cronbach's alpha for its subscales. The value of Cronbach's alpha ranges from 0 to 1, and a value closer to 1 is desirable and considered satisfactory. The Cronbach's alpha for QOLIE‐31 subscales was: *Seizure‐Worry* 0.74, *Emotional well‐being* 0.79*, Energy/Fatigue* 0.71, *Social Functioning* 0.76, *Cognitive‐Functioning* 0.81, *Medication‐effects* 0.81, and *Overall‐Quality of Life* 0.78. Thus, QOLIE‐31 has shown to be a reliable instrument for assessing HRQoL in PWE in the Pakistani population.

The Urdu version ofMARS was tested in a pilot study of 35 randomly selected “stable” epilepsy patients (controlled epilepsy), and the value of Cronbach's alpha was evaluated, which was 0.74, which shows satisfactory internal consistency. For test retest reliability, adherence was assessed after two weeks on 30 epilepsy patients. The value of the intraclass correlation coefficient of 0.75 (95% CI and *p* < .00) showed good reliability.

### 3.2 HRQoL of PWE

The mean (SD) total quality of life score of the seven domains of HRQoL among PWE was as follows: seizure worry (54.6 ± 21.5); overall quality of life (51.3 ± 13.2); emotional well‐being (48.6 ± 13.9); energy/fatigue (45.7 ± 16.1); cognitive function (54.5 ± 17.9); medication effects (55.9 ± 25.9); social function (50.4 ± 18.1). The overall weighted mean HRQoL score was (51.6 ± 17.1) Table 2. Table 3 shows HRQoL scores across sociodemographic characteristics of patients. Males (*p* ≤ .001), respondents of young age (*p* ≤ .001), those having higher levels of education (*p* ≤ .001), and married individuals (*p* ≤ .001), had better overall weighted HRQoL score. Additionally, respondents who could medicines (statistically insignificant) had less duration of epilepsy (*p* ≤ .001), were on monotherapy (*p* ≤ .001), and had better HRQoL scores.

**TABLE 2:** HRQoL and correlation with adherence of PWE in Pakistan (*n* = 219).

QOLIE‐31 subscales	Mean (SD)	95% Confidence interval	Pearson's correlation (HRQoL with adherence)
**Seizure worry**	54.600 (± 21.500)	51.780–57.460	0.930
**Overall quality of life**	55.900 (± 25.900)	52.490–59.330	0.910
**Emotional well‐being**	45.700 (± 16.100)	43.600–47.860	0.850
**Energy/fatigue**	48.600 (± 13.900)	46.730–50.410	0.840
**Cognitive function**	54.500 (± 17.900)	52.160–56.900	0.820
**Medication effects**	51.300 (± 13.200)	49.580–53.060	0.830
**Social function**	50.400 (± 18.100)	47.960–52.740	0.840
**Total score**	51.600 (± 17.100)	49.320–53.830	0.920

Significance 0.000.

**TABLE 3:** Means (SD) of adherence and HRQoL against characteristics of PWE.

Variables	Suboptimally adherent (*n*, %)	Adherent (*n*, %)	*p* Values	HRQoL (overall weighted mean score)	*p* Values
**Gender** ^a^			<.001		<.001
Male	47 (26.0)	69 (56.9)		63.7 (± 16.8)	
Female	67 (65.0)	36 (34.9)		51.4 (± 17.3)	
**Age groups** ^b^			<.001		<.001
18–25	27 (82.0)	45 (62.5)		55.8 (± 14.7)	
26–45	51 (48.1)	55 (51.9)		52.5 (± 17.1)	
>46	30 (73.1)	11 (26.8)		41.7 (± 17.1)	
**Education level** ^b^			<.001		<.001
Less than primary education	16 (80.0)	4 (20.0)		42.8 (± 18.9)	
Primary	6 (66.6)	3 (33.3)		44.1 (± 17.1)	
Secondary	86 (46.5)	99 (53.5)		52.6 (± 16.6)	
Tertiary	0	5 (100)		63.6 (± 2.40)	
**Marital status** ^a^					
Single	78 (54.2)	66 (45.8)		49.3 (± 17.5)	
Married/in relationship	30 (40.0)	45 (60.0)		56.0 (± 15.1)	
**Easy to afford medicines?** ^a^			.08		.07
Yes	48 (38.1)	78 (61.9)		56.7 (± 14.7)	
No	60 (64.5)	33 (35.5)		44.7 (± 17.6)	
**Duration of Epilepsy** ^b^			<.001		<.001
<5 years	64 (42.4)	87 (57.6)		53.7 (± 16.7)	
5–10 years	26 (66.7)	13 (33.3)		47.5 (± 16.3)	
>10 years	18 (62.1)	11 (37.9)		46.1 (± 18.1)	
**Antiepileptic drugs** ^a^			<.001		<.001
Monotherapy	19 (13.6)	121 (86.4)		52.8 (± 16.4)	
Combination therapy	18 (22.8)	61 (77.2)		49.4 (± 17.9)	

^a^ Independent samples *t*‐test.

^b^ One way ANOVA.

### 3.3 Adherence in PWE

The present study showed a total mean score for adherence 6.17 (± 2.31). Out of 219 respondents, 52.1% were suboptimal adherent (score 0–6), and 47.9% were adherent (score 7–10). Table 3 shows scores of adherence across different sociodemographic and clinical characteristics of PWE. Males (*p* ≤ .001), middle‐aged respondents (*p* ≤ .001), having higher levels of education (*p* ≤ .001), married (*p* ≤ .001), and those that could afford ASM medicines (statistically insignificant) had better adherence scores. Moreover, respondents with <5 years duration of epilepsy (*p* ≤ .001) and those on monotherapy (*p* ≤ .001) had better adherence.

### 3.4 Association of HRQoL and adherence in PWE

The relationship between HRQoL and medication adherence was investigated using Pearson's product‐moment correlation coefficient. The two variables were positively correlated and all scales of QOLIE‐31 showed a positive association with adherence (Table 2). The strongest association was found between adherence and seizure worry followed by emotional well‐being and energy/fatigue (Table 2).

### 3.5 Factors associated with HRQoL in PWE

The results for multiple linear regression analysis (unstandardized *β* coefficient; *p* value), after adjusting for covariates suggest medication adherence and employment status, are independent predictors for HRQoL (16.8; < 0.0001 and 7.5; 0.030). Additionally, males had significantly better overall HRQoL than females (10.0; 0.001). Moreover, educated patients, primary, secondary, and tertiary (0.780; < 0.0001, 0.820; 0.040, 0.910; 0.010), were associated with increased HRQoL score and respondents from age group ≥46 years (−0.680;  0.0001), duration of epilepsy >10 years (−0.700; <0.0001), and polypharmacy (−0.430; 0.010) were associated with low HRQoL score (Table 4).

**TABLE 4:** Multiple linear regression analysis for predicting variables for HRQoL domains in PWE in Pakistan.

QOLIE‐31 subscales									
Variable		Seizure worry *B*, SE, *β*, *p* value	Overall QOL *B*, SE, *β*, *p*value	Emotional WB *B*, SE, *β* ^2^, *p* value	Energy/fatigue *B*, SE, *β* ^2^, *p* value	Cognitive *β* ^1^ *B*, SE, *β* ^2^, *p* value	Medication Efficacy *B*, SE, *β* ^2^, *p* value	Social Function *B*, SE, *β* ^2^, *p* value	Overall Score *B*, SE, *β* ^2^, *p* value
**Level of adherence**									
	Adherent vs. nonadherent	14.800, 2.630, 0.180, <.0001	14.600, 2.180, 0.250, <.0001	12.745, 2.630, 0.154, <.0001	12.400, 3.335, 0.150 <.0001	10.155, 1.298, 0.280, <.0001	10.223, 3.350, 0.333, <.0001	12.600, 2.895, 0.180, <.0001	16.800, 3.890, 0.195, <.0001
	**Gender**								
1	(female vs. male)	–6.630, 2.361, −0.180, .001	–5.882, 1.835, −0.151, .040	–5.401, 1.250, −0.831, <.0001	–4.501, 1.944, −0.132, <.0001	–5.501, 1.145, −0.532, .002	–3.501, 1.191, −0.850, .001	–2.501, 0.451, −0.112, <.0001	–10.001, 1.940, −0.201, .001
	**Age**								
	26–45 years vs. 18–25 years	–0.530, 1.372, –0.051, .010	–0.450, 1.275, −0.251, .001	–0.441, 0.250, −1.250, <.0001	–0.470, 1.640, 0.025, <.0001	–0.270, 1.256, 0.010, <.0001,	–0.230, 0.250, −0.630, .001	–0.290, 0.100, −0.010, <.0001	–0.470, 2.101, −0.313, <.0001
	≥46 years vs. 18–25 years	–0.150, 1.721, −0.025, .920	–0.190, 1.040, −0.212, .020	–0.950, 2.330, −0.069, .023	–0.940, 0.673, −0.065, .001	–0.580, 0.450, –0.113, .020	–0.450, 1.201, −0.819, .002	–0.890, 1.250, 0.315, <.0001	–0.680, 2.240, −0.180, <.0001
	**Marital status**								
	Married vs. single	6.940, 2.925, 0.100, .048	0.023, 0.062, 0.020, .048	0.180, 1.042, 0.036, .007	0.200, 0.789. 0.025, .003	0.200, 1.020, 0.118, .003	0.140, 1.550, 0.225, .044	0.220, 1.230, 0.145, .001	0.190, 1.256, 0.1562, .005
	**Easy to afford medicines?**								
	No vs. Yes	–0.300, 1.115, 0.365, <.0001	–0.07, 1.360, −0.313, <.0001	–0.330, 1.344, −0.125, < .0001	–0.340, 1.256, −0.031, <.0001	–0.350, 2.925, −0.035, <.0001	–0.310, 1.365, −0.025, <.0001	–0.340, 1.389, −0.175, <.0001	–0.350, 1.200, −0.100, <.0001
	**Employment status**								
	Employed vs. unemployed	12.5, 2.562, 0.025, <.0001	0.310, 1.256, 0.025, .030	7.10, 1.25, 0.167, .003	7.10, 2.25, 0.037, .003	5.50, 2.958, 0.056, <.0001	6.50, 2.25, 0.313, <.0001	6.70, 1.36, 0.255, .030	7.50, 2.36, 0.112, .030
	**Education level**								
	Primary vs. illiterate	0.075, 0.226, 0.025, <.0001	0.830, 0.125, 0.033, <.0001	0.540, 0.258, 0.036, .001	0.500, 0.369, 0.056, <.0001	0.620, 0.258, 0.001, <.0001	0.620, 0.125, 0.036, <.0001	0.450, 1.325, 0.036, .002	0.780, 1.005, 0.360, <.0001
	Secondary vs. illiterate	0.105, 0.356, 0.001, .001	0.700, 0.658, 0.056, .020	0.450, 2.285, 0.031, .001	0.750, 1.895, 0.025, .020	0.930, 1.695, 0.098, .300	0.780, 0.145, 0.168, <.0001	0.410, 0.254, 0.036, <.0001	0.820, 2.004, 0.015, .040
	Tertiary vs. illiterate	0.711, 0.589, 0.014, .010	0.600, 1.250, 0.368, .020	0.440, 1.360, 0.313, .101	0.500, 2.260, 0.056, .011	0.780, 1.452, 0.015, .040	0.580, 2.365, 0.047, <.0001	0.500, 1.365, 0.056, <.0001	0.910, 1.257, 0.011, .010
	**Duration of epilepsy**								
	5–10 years vs. < 5 years	–0.150, 0.578, −0.002, <.0001	–0.156, 1.058, −0.005, .001	–0.130, 1.008, −0.007, .010	–0.120, 2.368, −0.058, <.0001	–0.900, 1.365, 0.045, .100	–0.800, 1.354, −0.004, .100	–0.200, 2.378, 0.115, <.0001	–0.404, 1.365, −0.0313, .100
	>10 years vs. < 5 years	–0.640, 1.025, −0.005, <.0001	–0.840, 1.225, −0.004, .001	–0.420, 2.058, −0.004, .010	–0.310, 1.157, −0.008, <.0001	–0.100, 1.369, −0.036, .010	–0.400, 1.895, −0.003, .010	–0.85, 1.250, −0.133, <.0001	–0.700, 1.112, −0.002, <.0001
	**ASM medication**								
	Combination therapy vs. Monotherapy	–0.350, 2.240, −0.004, .010	–0.330, 1.036, −0.007, .010	–0.380, 1.025, −0.012, <.0001	–0.370, 1.005, −0.007, .001	–0.400, 1.225, −0.115, .040	–0.390, 1.365, −0.115, .001	–0.460, 1.589, −0.008, < .0001	–0.430, 1.367, −0.117, .010
	***R* ^2^, Adjusted *R*** ^2^	0.894, 0.892	0.797, 0.782	0.270, 0.290	0.350, 0.390	0.450, 0.420	0.262, 0.284	0.570, 0.580	0.680, 0.690

## 4 DISCUSSION

In this study, majority of PWE had suboptimal adherence and a HRQoL score of 51.6 (± 17.1), and an association was found between adherence and HRQoL. The current research show various independent factors are associated with adherence and HRQoL of PWE, such as gender, duration illness, level of education, and employment status.

The QOLIE‐31 total score was 51.6 (± 17.1) in the present study, congruent to research conducted a decade ago in a small number of PWE (*n* = 50) in Pakistan (Najam‐Us‐Sahar & Chaudry, 2010). Similarly, studies conducted on PWE from India, Turkey, and Iran reported a total mean score of 60.8 (± 17.03) (Mehndiratta et al., 2015), 56.4 (± 17.3) (Mollaoglu et al., 2015), and 53.4 (± 22.8) (Mohammadi et al., 2013) respectively, which are similar to our findings. Similar studies on PWE from Benin and Uganda report similar QOLIE‐31 scores: 52.1 (± 33.4) (Nubukpo et al., 2004) and 58.1 (± 13.1) (Nabukenya et al., 2014). However, studies from Nigeria and Ethiopia report much higher QOLIE‐31 scores than our findings: 77.98 (± 13.32) (Ogundare et al., 2021) and 86.5 (± 24.31) (Kassie et al., 2021), respectively. Our study's lower QOLIE‐31 score may be partially explained by differences in sociodemographic factors, sample size variations, healthcare quality, and study environment (Tefera et al., 2020). In contrast, our results are better than findings from Russia that have a QOLIE‐31 score of 48 (± 10.08), which might be because the study population consisted of disabled elderly PWE (Melikyan et al., 2012). Regarding the HRQoL score of the seven domains of QOLIE‐31, respondents scored highest in the medication effects domain and scored lowest on the energy/fatigue domain. A consistent finding that respondents have scored lowest in energy/fatigue domain was reported by the first study conducted in PWE in Pakistan (Najam‐Us‐Sahar & Chaudry, 2010) and a similar study conducted in PWE from Northeast Ethiopia (Kassie et al., 2021); this indicates that respondents were feeling fatigue, tiredness and low energy levels. Another study reported respondents scored highest in medication effects domain in PWE (Gebre & Haylay, 2018) and also in studies conducted in Malaysia (Norsa'adah et al., 2013) and West Africa (Nubukpo et al., 2004). This indicates that PWE in current study have experienced low physical and mental side effects associated with ASM therapy, which may be explained by changes in sample size characteristics or environment. A study from Seattle, USA, reported a mean score of 58 (± 16) (Baca et al., 2015), which is similar to current study. The same study scored somewhat higher across all domains in comparison to our study which again may be explained by differences in sample size and increased access to latest diagnostic and treatment services. These findings are congruent with two studies from Europe (Spain) with scores of 61.8 (± 17.3) (Villanueva et al., 2013) and 70.8 (± 13.3) (Yadegary et al., 2015), which have slightly higher scores across all domains than the present study. Similarly, studies from Hungary (Lám et al., 2001), France (Picot et al., 2004), Greece (Haritomeni et al., 2006), Czech Republic (Tlusta et al., 2009), and Japan (Kubota & Awaya, 2010) report higher scores than current study, that is, 55.5 (± 19.3), 61.9 (± 19.0), 68.5 (± 17.2), 65.6 (± 18.3), and 60.9 (± 30.5). The reason for lower score from Pakistan may be due to stark differences in healthcare facilities, decreased disease awareness, and limited access to proper diagnostic facilities.

Male sex was associated with increased scores of HRQoL, which is in line with findings of studies in PWE from Northeast Ethiopia (Kassie et al., 2021) and Portugal (Silva et al., 2019). In India, it was also reported that female gender was associated with decreased levels of HRQoL among PWE (Ashwin et al., 2019). This may be because many women in Pakistan, especially in rural areas, experience stigma, restriction on attending family functions and receiving education, and face challenges in being employment and getting married (Bhalla et al., 2017; Lalwani et al., 2015). Having higher levels of education was associated with increased levels of HRQoL in PWE. The finding that higher education is associated with better HRQoL in PWE is supported by studies from Uganda (Nabukenya et al., 2014), Ethiopia (Kassie et al., 2021), and Kenya (Kinyanjui et al., 2013). This might be because educated patients may have better social support, higher confidence and self‐esteem, a better understanding of their medical condition, and more awareness on the importance of medication adherence and its impact on HRQoL (Martínez et al., 2008).

The findings of the present study show that older PWE (≥46 years) have worse overall HRQoL, which is congruent with findings reported from other studies (Edefonti et al., 2011; Michelson et al., 2000). A prolonged duration of illness (>10 years) has shown to be an independent predictor of low HRQoL in our study, this is consistent with studies conducted in Italy and Spain (Benavente‐Aguilar et al., 2004; Edefonti et al., 2011). As the duration of epilepsy and age increases, the overall cognitive function declines, which contribute to poor HRQoL (Edefonti et al., 2011; Jokeit & Ebner, 2002), which may explain low HRQoL reported in present study in PWE (≥46 years) and duration of illness (>10 years).

Employed individuals had increased levels of HRQoL, which is congruent to studies from Saudi Arabia (Altwijri et al., 2021) and China (Gu et al., 2016). This may be because occupational status reflects socioeconomic status, and low socioeconomic status is associated with low HRQoL (Hesdorffer et al., 2005). Regarding marital status, married individuals had better HRQoL than single PWE; this finding is consistent with a study from Uganda (Nabukenya et al., 2014). This may be because married individuals may have more social and psychological support.

Regarding ASM therapy, polypharmacy was an independent predictors of decreased HRQoL in PWE in current study and this finding is in line to other studies on PWE (Bautista & Tannahill Glen, 2009; Iwuozo et al., 2020; Kinyanjui et al., 2013; Thomas et al., 2005). The present study found a significant correlation between ASM adherence and HRQoL, which has also been suggested in other studies conducted on PWE (Eatock & Baker, 2007; Hovinga et al., 2008). This is because adherence to ASM therapy is associated with decreased seizure frequency and consequently have increased levels of HRQoL.

Regarding adherence, the present study had a mean adherence score of 6.1 (± 2.31) with 52.1% of patients found to have suboptimal adherence to ASMs, which is a proportion significantly higher than reported by a study in Ethiopia with a nonadherence rate of 37.80% (Getnet et al., 2016). In contrast, studies from Finland, North Carolina, and the United Kingdom found lower nonadherence rates 34%, 39 %, and 36.40% (Chapman et al., 2014; Davis et al., 2008; Kyngäs, 2000), and a study from Brazil reported a higher rate (66.20%) than the present study (Ferrari et al., 2013). Ferrari et al. (2013) found that multidrug treatment was associated with lower adherence rates. The lower adherence rate in the current study may be explained by differences between developed and developing countries in terms of literacy rates, management and quality of healthcare services, and ease of access to medications (Cárdenas‐Valladolid et al., 2010). In Pakistan, similar nonadherence rates have been reported in hypertensive patients (Ahmed et al., 2021; Saqlain et al., 2019), asthmatic patients (Ahmed et al., 2017), and cardiovascular disease patients (Saqlain et al., 2021). This may be due to improper counseling (Ahmed et al., 2018) because of an overburdened healthcare setup, low annual income, lack of awareness about disease management (Kamal et al., 2016; Qidwai, 2009), unavailability and higher price of medications, and lack of insurance policies (Klara & Kiani Ayyaz, 2006). Males had higher adherence and HRQoL in our study than females, which was consistent with Portuguese (Silva et al., 2019), Indian (Ashwin et al., 2019), and Ethiopian study (Kassie et al., 2021). This finding is not limited to epileptic patients, and Ahmed et al. (2021) reported HIV‐positive Pakistani females had low medication adherence and consequently a decreased HRQoL. As medication adherence is an independent predictor of HRQoL, low adherence among females has been reported in other diseases (Khayyat et al., 2019). This partially may be due to hormonal changes in response to ASMs in females (Michelson et al., 2000; Yadegary et al., 2015), male societal dominance and higher education levels, lack of freedom, and higher physical and psychological stress among females (World Medical Association, 2013). Patients on monotherapy had significantly better adherence and increased levels HRQoL than the ones on combination therapy; this is consistent with other studies (O’ Rourke & O’ Brien, 2017; Zafar et al., 2019) and maybe because of increased side effects to ASMs or treatment ineffectiveness due to resistant epilepsy, which may subsequently reduce adherence and consequently HRQoL (Khayyat et al., 2019).

### 4.1 Limitations

The present study had certain limitations. Self‐reported measures are frequently associated with recall bias and the use of such measures in the present study might have been responsible for misestimating the true incidence of adherence and subsequently its impact on and association with HRQoL. Notably, we cannot ascertain the direction of adherence and HRQoL association (whether it was poor adherence that led to poor HRQoL or vice versa) because this was a cross sectional study; therefore, future cohort or randomized controlled trials are needed to confirm the direction of the association. A self‐report measure of adherence was used in this study because of lack of funding to support other robust measures of adherence like serum medication levels. The respondents who had less than primary education (*n* = 5, 2.30%) were verbally interviewed, and this may have been responsible for social desirability bias when asking about adherence. Depression and anxiety are strong predictors of HRQoL and affect adherence, and the current study does not utilize them as covariates, which limits the accuracy of the results.

Lastly, this study used an Urdu version of QOLIE‐31 and MARS; thus, only those PWE were selected that could converse and understand Urdu. Consequently, the results of our study cannot be generalized to non‐Urdu speaking populations of Pakistan but because Urdu is the national language of Pakistan so majority of the population in urban areas (this study conducted in an urban area) can understand and converse in Urdu.

## 5 CONCLUSION

Overall, PWE in our centers had better HRQoL if they were adherent to ASMs. Medication adherence and HRQoL had a significant correlation across all items and scales of MARS and QOLIE‐31, respectively. Female gender, age group (≥46 years), prolonged duration of epilepsy (>10 years), low education, polypharmacy, and unemployment were significant predictors of poor adherence and consequently poor HRQoL; thus, such factors need special attention in designing evidence‐based interventions. The findings from the current study can be used to support devising population‐specific interventions to improve adherence and HRQOL for PWE in Pakistan. For future studies, an important consideration could be to include a subset of medically refractory epilepsy patients who are not adherent because their symptoms did not improve from ASM therapy. Identifying this subgroup and understanding factors pertaining to their HRQoL will improve our understanding of PWE in our region.

## AUTHOR CONTRIBUTIONS

M.T. was responsible for conceiving and conceptualizing the idea of the research. M.T., A.H.T, M.I., F.A., and A.A. designed and developed the study protocol. M.T. and A.A. collected the data. M.T., A.A., and A.H.T. analyzed the data. M.T. and A.H.T wrote and generated the first draft of the manuscript. Revision was done by M.T. and A.H.T. Subsequently, the first draft was reviewed by M.I., F.A., A.H.T, and A.A. The manuscript was approved by all authors.

## CONFLICT OF INTEREST STATEMENT

The authors declare that they have no competing interest.

## ETHICS STATEMENT

Ethical approval for the conduct of the study was obtained from the Ethical Review Board of Pakistan Institute of Medical Sciences (PIMS) and Rawalpindi Medical University (RMU), Pakistan (Approval No;1255, 1028). All procedures performed in the study were by good clinical practice and the Declaration of Helsinki 1964 and comparable ethical standards. Written informed consent was obtained from all the participants involved in this study.

### PEER REVIEW

The peer review history for this article is available at https://publons.com/publon/10.1002/brb3.3127

## Data Availability

The data sets used in this study can be requested from the corresponding author if justified.
